# Coix Seed Attenuates Acute Lung Injury via NLRP3 Signaling Pathway: Efficacy, Active Components, and Mechanisms

**DOI:** 10.1002/fsn3.72327

**Published:** 2026-09-06

**Authors:** Qi Yan, Yuyan Li, Tingyan Chen, Wenjing Liu, Youshuo Dong, Zhangbo Ao, Hongmei Wu, Feng Xu

**Affiliations:** ^1^ Department of Pharmacy Guizhou University of Traditional Chinese Medicine Guiyang China; ^2^ Research Center for Coix Seed Resources and Development Guizhou University of Traditional Chinese Medicine Guiyang Guizhou China

**Keywords:** acute lung injury, bioactive compounds, Coix seed, network pharmacology, NLRP3 signaling pathway

## Abstract

Coix seed is a medicinal and nutritional resource, but its protective effects against acute lung injury (ALI) remain unclear. This study investigated the efficacy, active components, and mechanisms of Coix seed extract (CSE) against ALI, focusing on the NLRP3 signaling pathway. The therapeutic efficacy of CSE was evaluated in a murine model of ALI. Liquid chromatography‐mass spectrometry identified 32 components in CSE, which were then analyzed via network pharmacology, molecular docking, and molecular dynamics simulations for target prediction and drug‐likeness evaluation. In vivo effects on NLRP3 pathway proteins were also examined. CSE decreased serum levels of TNF‐α and IL‐1β, pulmonary levels of IL‐1β and IL‐18, indicating systemic and local inflammatory suppression. Furthermore, CSE downregulated the expression of NLRP3, Caspase‐1, and GSDMD‐N in lung tissues, suggesting inhibition of NLRP3 inflammasome‐driven pyroptosis. Network analysis screened 35 core targets and 4 NLRP3‐related key targets involved in pathways regulating inflammation, proliferation, and oxidative stress. Molecular docking and dynamics simulations further corroborated the “component‐target‐disease” interactions within this network. Drug‐likeness evaluation identified 12 promising compounds, including syringaldehyde, tangeretin, and eriodictyol. Collectively, these findings demonstrate that CSE alleviates ALI by suppressing both inflammation and pyroptosis through multiple pathways, with NLRP3 as a central mechanistic hub. The identification of bioactive compounds with favorable pharmacokinetic properties provides a chemical basis for quality control and supports potential application of CSE as a functional food ingredient in the management of pulmonary inflammatory conditions.

## Graphical abstract

From component identification to target validation: Coix seed extract exerts a protective effect against acute lung injury (ALI) by synergistically inhibiting inflammatory cytokines and NLRP3‐mediated pyroptosis through multiple signaling pathways.

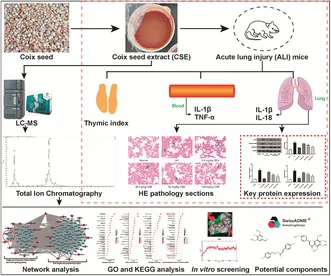

## 1 Introduction

Acute lung injury (ALI) is a severe clinical condition characterized by alveolar damage, impaired gas exchange, and hypoxemia, often progressing to acute respiratory distress syndrome (ARDS) with mortality rates ranging from 30% to 40% (Gu et al. 2024). Despite decades of research, pharmacological interventions remain largely supportive, and no specific targeted therapy has been approved for ALI, primarily due to its complex and multifactorial pathophysiology, which involves uncontrolled inflammation, oxidative stress, and alveolar epithelial cell injury (Tian et al. 2024). Historically, the pathophysiological hallmarks of ALI have included diffuse alveolar damage and increased pulmonary vascular permeability, as initially established in landmark experimental models (Ware and Matthay 2000). These earlier findings laid the groundwork for understanding the central role of alveolar–capillary barrier disruption in disease progression. Even with recent progress in treating ALI and its subsequent stage, ARDS, overall morbidity and mortality remain high (Gu et al. 2024). Sepsis is a critical infectious disease, and ALI is the most immediate and severe complication arising from it (Borges and Bento 2024). This association has been well documented since the early descriptions of sepsis‐induced lung injury, wherein endotoxin‐mediated systemic inflammation was identified as a key driver of acute respiratory failure (Bernard et al. 1994; Hudson et al. 1995). Recently, traditional Chinese medicine has demonstrated potential applications in ALI treatment and is gradually gaining attention (Liu, Wang, et al. 2024). In this context, dietary phytochemicals derived from medicinal plants have been increasingly recognized for their multifaceted roles in disease prevention and management, with growing evidence supporting their anti‐inflammatory and antioxidant properties via various molecular mechanisms (Hossain et al. 2025a).

The dried mature seed kernel of *Coix lachryma‐jobi* L. var. *ma‐yuen* (Roman.) Stapf, commonly known as Coix seed, is a traditional Chinese herb (Chinese Pharmacopoeia Commission 2020). The traditional therapeutic effects of Coix seed include eliminating dampness, promoting urination, and strengthening the spleen to prevent diarrhea, alleviate arthritic pain, remove pus, and diminish stagnation via detoxification (Chinese Pharmacopoeia Commission 2020). Coix seed oil can attenuate radiation‐induced lung injury through its anti‐inflammatory effects (Zhu et al. 2022). Historically, Coix seed was documented in traditional Chinese medicine classics, such as the *Compendium of Materia Medica* (Li Shizhen, Ming Dynasty), for its properties of expelling pus and relieving pulmonary suppuration. Additionally, it is recorded in the Fan Wang Formula for the treatment of lung abscesses. Patients with severe lung abscesses typically present with high fever, dyspnea, and hypoxemia—clinical manifestations that resemble the acute respiratory failure seen in ALI, suggesting a potential therapeutic overlap. Furthermore, Coix seed extract (CSE) possesses anti‐inflammatory and immunomodulatory properties (Chung et al. 2021; Seo et al. 2000), providing a rationale for its potential application in sepsis‐induced ALI. The interplay between dietary components and health outcomes is well established, with emerging evidence highlighting the importance of phytochemical‐rich foods in modulating chronic inflammatory conditions and supporting immune function (Hossain et al. 2025). Accordingly, CSE prepared using 45% ethanol may exert protective effects against ALI. However, the active components and underlying mechanisms of CSE in the prevention of ALI remain to be elucidated.

The NLRP3 inflammasome is a key mediator of inflammatory responses in ALI. Upon activation, it promotes the release of IL‐1β and IL‐18 and facilitates the cleavage of gasdermin D (GSDMD) to induce pyroptosis, a form of programmed cell death that exacerbates barrier disruption, oxidative stress, and tissue damage (Gu et al. 2024). These observations have made NLRP3 an attractive target for anti‐inflammatory interventions. However, the clinical development of synthetic NLRP3 inhibitors has faced obstacles, including safety concerns and the protein's role in normal immune functions, making its complete blockade potentially problematic. Therefore, natural products derived from medicinal and nutritional sources offer an alternative strategy, as their multicomponent nature may allow for partial or balanced modulation of inflammatory pathways with a more favorable safety profile.

Coix seed, a well‐documented anti‐inflammatory agent in both traditional medicine and modern pharmacological studies, represents a promising candidate in this regard. Therefore, investigating its protective mechanisms via the NLRP3 pathway is both mechanistically grounded and practically relevant to its potential application as a functional food ingredient for pulmonary health. In addition, network analysis illustrates the multifaceted effects of functional foods across various components, targets, and pathways; these predictions can be corroborated via molecular docking and molecular dynamics (MD) simulations, thereby helping to elucidate their underlying mechanisms of action (Wu et al. 2024). Specifically, network pharmacology analysis helps clarify the mechanism by which CSE alleviates ALI. Moreover, the relationship between dietary choices, nutrition, and overall health has become a central theme in modern nutritional science, emphasizing the role of diet‐derived bioactive compounds in maintaining physiological homeostasis and mitigating disease risk (Hossain et al. 2025b).

In this work, employing an integrated approach comprising network pharmacology, molecular docking, MD simulations, and in vivo validation, the present study first utilized a lipopolysaccharide (LPS)‐induced murine model of ALI to assess the therapeutic efficacy of CSE. Subsequently, liquid chromatography‐mass spectrometry (LC‐MS) was employed to identify the components of CSE and to comprehensively analyze its mechanisms against ALI using network pharmacology. Next, molecular docking and MD simulations were used to corroborate the reliability of the network analysis predictions, and the drug‐likeness properties of the identified compounds were evaluated. Finally, the regulatory effects of CSE on the NLRP3 signaling pathway were evaluated in vivo. Ultimately, this study provides a pharmacological basis for the use of Coix seed in the treatment of ALI and supports its continued development as a functional therapeutic agent.

## 2 Materials and Methods

### 2.1 Drugs and Reagents

Coix seed, acquired from the Taisheng Traditional Chinese Medicine Specialty Market (Guiyang, China), was authenticated by Professor Xiangpei Wang (Guizhou Minzu University) as the mature seed of *Coix lachryma* jobi L. The following reagents were utilized: LPS (Sigma‐Aldrich Trading Co. Ltd., 0000263267); mouse TNF‐α (Ltd, M241015‐102a), IL‐1β (M241015‐001b), and IL‐18 (M241015‐011) ELISA kits (Xinbosheng Bio); dexamethasone (DEX, used as a positive control) (Huazhong Pharmaceuticals Co. Ltd., H42041292).

### 2.2 Preparation of CSE

Moderate ethanol concentrations (40%–50%) efficiently extract both polar and moderately non‐polar bioactive compounds from Coix seed, including flavonoids and phenolic acids. Coix seeds (2 kg) were extracted three times with 45% (v/v) ethanol. For each extraction, 12,000 mL of 45% ethanol was added to the seeds; the mixture was soaked for 15 min, heated to boiling, and allowed to reflux for 1 h. During extraction, the solution transitioned from light yellow to slightly milky white. After filtration, the three filtrates were combined to yield the crude CSE solution. The combined extract was concentrated under reduced pressure using a rotary evaporator to remove the ethanol, and the resulting concentrate was further evaporated to dryness in a water bath at 85°C to eliminate residual solvent, yielding CSE. High‐performance liquid chromatography (HPLC) fingerprinting was employed for the batch‐to‐batch quality control of CSE, ensuring the stability and reproducibility of the product (Figure S1).

### 2.3 Animals

This study utilized male Kunming mice (20–25 g), supplied by the Institute of Experimental Animals at Guizhou University of Traditional Chinese Medicine (license No. SCXK [QIAN] 2021–00055). Male animals were selected to minimize the confounding effects of the estrous cycle on inflammatory responses and to facilitate intra‐group comparability. This approach is consistent with previous ALI studies that utilized a single‐sex design for initial mechanistic exploration (Xu et al. 2017b). All animals were maintained under standardized conditions (temperature: 22°C ± 2°C, humidity: 50%–60%, 12‐h light/dark cycle) with *ad libitum* access to standard chow and water. Ethical approval for the animal experiments was granted by the Research Ethics Committee of Guizhou University of Traditional Chinese Medicine University (Reference No. 20240831001; approved August 31, 2024).

### 2.4 Animal Grouping, Modeling, and Treatment

Sixty mice were randomly assigned to a normal group, an ALI model group, a DEX‐positive control group (5 mg/kg), and three CSE treatment groups (26, 52, and 104 mg/kg), with 10 animals per group. The administered dose of CSE was calculated based on the human clinical dose of 30 g/day of raw Coix seed, utilizing body surface area (BSA) normalization and adjusting for the extraction yield of CSE. The resulting equivalent mouse dose was 52 mg/kg body weight. The dose of DEX was derived from the human clinical dose via BSA‐based allometric scaling (Xu et al. 2017b). The CSE was administered via oral gavage to the three treatment groups, while the normal and ALI model groups received an equivalent volume of saline. These treatments were administered consecutively for 7 days. The positive control group received an intraperitoneal injection of DEX (5 mg/kg) on Days 6 and 7 (Xu et al. 2017b). On Day 7, the animals were fasted for 12 h. Thirty minutes post‐treatment, mice in the ALI, DEX, and CSE groups received an intraperitoneal injection of LPS (15 mg/kg) to induce modeling, whereas the normal group received an equivalent volume of saline (Xu et al. 2017b). Twelve hours post‐LPS injection, the mice were euthanized for tissue and blood collection to measure the relevant indices.

### 2.5 Organ Indices, Lung Wet/Dry Ratio, and Tissue Collection

Following anesthesia, blood was collected via retro‐orbital bleeding, and the serum was separated and stored. Subsequently, the animals were euthanized via decapitation. The spleen, thymus, lungs, heart, liver, and kidneys were excised and weighed to calculate the organ indices. A portion of the right lung was weighed and subsequently dried in an oven at 68°C for 48 h. The lung wet/dry (W/D) ratio was calculated as the wet weight (g) divided by the dry weight (g), multiplied by 100% (Song et al. 2000). A portion of the lung tissue was fixed in 4% paraformaldehyde, while the remainder was snap‐frozen in liquid nitrogen and stored at −80°C.

### 2.6 Histopathological Evaluation of Lung Tissues

Following fixation in 4% paraformaldehyde for 48 h, the lung tissues underwent standard processing, including ethanol dehydration, clearing in xylene, paraffin embedding, and sectioning. The sections were stained with hematoxylin‐ and eosin (H&E) and examined under a light microscope with representative images captured (Xu et al. 2017a). Lung tissue injury was scored according to a five‐point scale based on the severity of pathological alterations: 0 (normal), 1 (minimal), 2 (mild), 3 (moderate), and 4 (severe). The scoring criteria focused on three key parameters: interstitial inflammatory cell infiltration, hyperemia, and type II alveolar epithelial hyperplasia.

### 2.7 Assessment of Inflammation‐Related Factors in Serum and Lung Tissue

Serum levels of TNF‐α and IL‐1β were assessed via enzyme‐linked immunosorbent assay (ELISA), strictly following the manufacturer's instructions. Additionally, lung tissues were homogenized and centrifuged to isolate the supernatants. The pulmonary expression levels of IL‐lβ and IL‐18 were subsequently quantified using the respective ELISA kits.

### 2.8 Identification of Components in CSE

The CSE samples were analyzed using an ultra‐performance liquid chromatography‐electrospray ionization‐tandem mass spectrometry (UPLC‐ESI‐MS/MS) system consisting of a Waters Acquity I‐Class PLUS UPLC coupled to an Applied Biosystems QTRAP 6500^+^ mass spectrometer. Chromatographic separation was performed on a Waters HSS‐T3 UPLC column (1.8 μm, 2.1 mm × 100 mm) maintained at 50°C. The mobile phase comprised solvent A (water with 0.1% formic acid and 5 mM ammonium acetate) and solvent B (acetonitrile with 0.1% formic acid). The flow rate was set at 0.35 mL/min, and the injection volume was 4 μL. The gradient elution program was as follows: 0–1.5 min, 98% A and 2% B; 1.5–5.0 min, linear gradient to 50% A and 50% B; 5.0–9.0 min, gradient to 2% A and 98% B, held for 1 min; then returned to 98% A and 2% B within 1 min and equilibrated for 3 min.

The ESI source parameters were set as follows: source temperature, 550°C; ion spray voltage, +5500 V for positive ion mode and −4500 V for negative ion mode. The collision‐activated dissociation (CAD) was set to a moderate level. Instrument tuning and mass calibration were performed using polypropylene glycol solutions at concentrations of 10 μmol/L (for triple quadrupole [QQQ] mode) and 100 μmol/L (for linear ion trap [LIT] mode), respectively. A mass tolerance of 5 ppm, along with literature and MS/MS fragmentation data, guided compound identification.

### 2.9 Target Prediction for CSE Components and ALI

Based on the components identified via LC‐MS, the potential targets of the active compounds were predicted using the SwissTargetPrediction platform (screening criterion: probability > 0). Disease‐associated targets for ALI were retrieved from the GeneCards, OMIM, and TargetNet database. For the GeneCards database, the screening criterion was set to a relevance score > 25 to filter for targets significantly associated with ALI. The data from these three databases were subsequently merged to establish the final target dataset for ALI.

### 2.10 Construction of the “Component‐Target‐Disease” Network

To construct the “component‐target‐disease” network, the target sets of the CSE components and ALI were intersected to obtain common targets. The resulting common targets were subjected to PPI network analysis using the STRING database, and species were set to *Homo sapiens* , and the confidence score threshold was set to 0.9 (highest confidence). Finally, the component‐target associations, disease‐target associations, and the PPI network were integrated and visualized using Cytoscape software. Five algorithms within the CytoHubba plug‐in were utilized for topological analysis, and core targets were identified as nodes with maximal clique centrality (MCC), maximum neighborhood component (MNC), degree, betweenness, and closeness values at or above the median for all network nodes (Yu et al. 2023).

### 2.11 GO and KEGG Enrichment Analyses

The R programming language was used to conduct GO and KEGG pathway enrichment analyses on the core targets. First, the genes were annotated using the org.Hs.eg.db data package. Subsequently, enrichment analyses were performed using the colorspace and stringi packages, alongside Bioconductor packages such as DOSE, clusterProfiler, and pathview (Xu et al. 2025).

### 2.12 Molecular Docking Screening of NLRP3 Pathway Related Targets

Based on the CytoHubba topological analysis, the core targets with the top two‐degree values, along with key targets related to the NLRP3 signaling pathway, were selected for molecular docking validation. The 2D structural formulas of the CSE components were retrieved from the TCMSP or PubChem databases, and the 3D structures of the core targets (in PDB format) were obtained from the RCSB Protein Data Bank (PDB). Protein preparation, including the removal of solvent and organic molecules and the addition of hydrogen atoms, was performed using PyMOL software; subsequent docking analyses were executed using AutoDock Vina (Xu et al. 2025). The docking grid box sizes and coordinate information were as follows: STAT3 (PDB ID: 6NJS; coordinates: 1.577, 48.614, 7.682; size: 48 × 66 × 52), AKT1 (PDB ID: 3QKM; coordinates: 20.605, 2.325, 16.232; size: 48 × 52 × 58), NLRP3 (PDB ID: 8WSM; coordinates: 14.468, 31.766, 1135.723; size: 40 × 40 × 40), RELA (PDB ID: 1NF1; coordinates: −4.049, 51.25, −4.205; size: 40 × 56 × 108), and CTSB (PDB ID: 8HE9; coordinates: −7.617, 0.262, 8.224; size: 44 × 50 × 52).

### 2.13 MD Simulations

To evaluate the binding stability between the screened compounds and targets, the eriodictyol‐AKT1 complex—which exhibited the highest molecular docking affinity—was selected for study, and MD simulations were performed using GROMACS version 2023.2. The CHARMM36 force field and CHARMM‐modified TIP3P water model were utilized for the simulation. Following preprocessing including the definition of the simulation box, solvation, ion addition, charge neutralization (via the Particle Mesh Ewald method), energy, minimization, constraint imposition, and sequential NVT (at 300 K), and NPT (at 1 bar) equilibrations—the 100‐ns MD simulations were conducted. Parameters including root mean square deviation (RMSD), root mean square fluctuation (RMSF), and the radius of gyration (Rg) of the protein‐ligand complexes were subsequently extracted and analyzed.

### 2.14 Evaluation of Gastrointestinal Absorption and Drug‐Likeness Properties

The gastrointestinal (GI) absorption and drug‐likeness of the components within the “component‐target‐disease” network were evaluated using the SwissADME website platform (Daina et al. 2017). The screening criteria were defined as follows: high GI absorption, compliance with Lipinski's rule of five (Lipinski et al. 2001), and at least three “Yes” responses across the Ghose, Veber, Egan, and Muegge indicators.

### 2.15 Determination of Key NLRP3 Pathway Protein Expression via Western Blot

Lung tissue samples from each group were pulverized in liquid nitrogen, homogenized in lysis buffer and incubated on ice. The homogenates were subsequently centrifuged to isolate the protein supernatants, and the protein concentrations were quantified by BCA assay and adjusted to equal concentrations prior to loading, ensuring identical total protein amounts per well (Xu et al. 2017b). The target proteins and GAPDH were detected on the same membrane using a sequential probing protocol. The primary antibodies for NLRP3, Caspase‐1, GSDMD‐N, and GAPDH were diluted at ratios of 1:1000, 1:1000, 1:2000, and 1:10,000, respectively. Relative protein expression was determined by calculating the ratio of the grayscale value of the target protein bands to that of the internal reference protein (GAPDH) bands.

### 2.16 Data Analysis

Data are expressed as the mean ± error of the mean (SEM). Statistical analyses were performed using SPSS Statistics v25.0 (IBM Corp., Armonk, NY, USA). Normality was assessed using the Shapiro–Wilk test, and the homogeneity of variances was evaluated using Levene's test. For data meeting both assumptions, a one‐way ANOVA was conducted, followed by LSD post hoc tests for multiple comparisons. When the homogeneity of variance assumption was violated, Dunnett's T3 test was used for post hoc multiple comparisons. A value of *p* < 0.05 was considered statistically significant. A post hoc power analysis was performed using GPower 3.1.9.7 based on the primary outcomes (IL‐1β and IL‐18). Using the observed partial *η* ^2^ values from the one‐way ANOVA (IL‐1β: 0.85; IL‐18: 0.78), with *α* = 0.05, six groups, and *n* = 10 per group, the achieved power exceeded 0.99 for both indicators, verifying that the sample size was adequate for the main analyses.

## 3 Results

### 3.1 Organ Indices and Lung Wet/Dry Ratio

**FIGURE 1:**
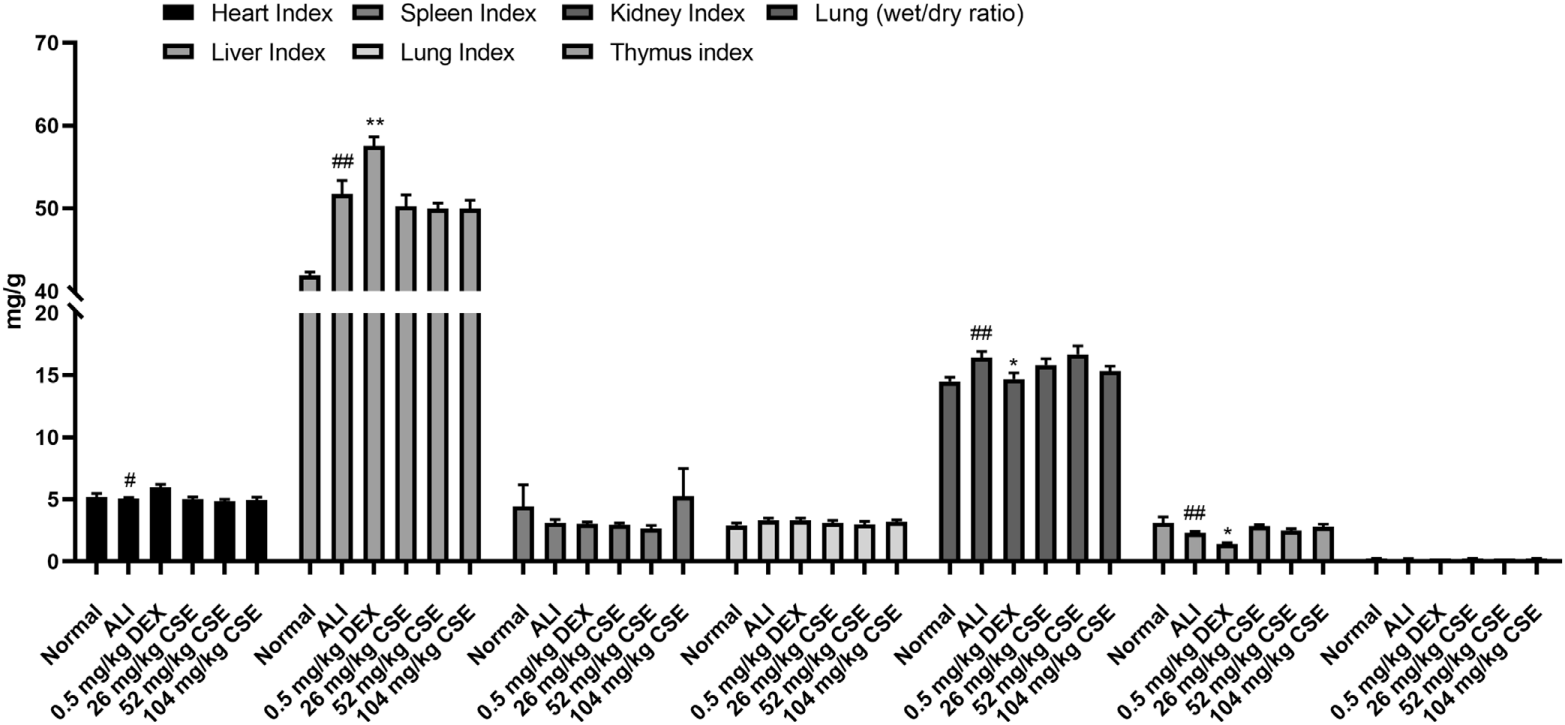
Changes in heart, liver, spleen, lungs, kidneys, thymus index and lung wet/dry ratio in various groups of animals. Data are presented as mean ± SEM (*n* = 10). ^#^ *p* < 0.05 versus the control group, ^##^ *p* < 0.01 versus the normal group, *p < 0.05 versus the ALI group, **p < 0.01 versus the ALI group.

The liver and kidney indices in the ALI group were significantly higher than those in the normal group (*p* < 0.01). In contrast, the cardiac index in the ALI group was significantly lower than that in the normal group (*p* < 0.05). CSE treatment exhibited a trend toward improvement in the heart, liver, lung, spleen, kidney, and thymus indices, as well as the lung wet/dry ratio in ALI mice, although none of these changes reached statistical significance (*p* > 0.05) (Figure 1). Although CSE treatment did not significantly reverse these changes, the consistent trend toward improvement across all measured organ indices and the lung wet/dry ratio suggests a potential systemic protective effect that may require a larger sample size or extended treatment duration to reach statistical significance.

### 3.2 Histopathological Evaluation of CSE Effects on Lung Tissues

**FIGURE 2:**
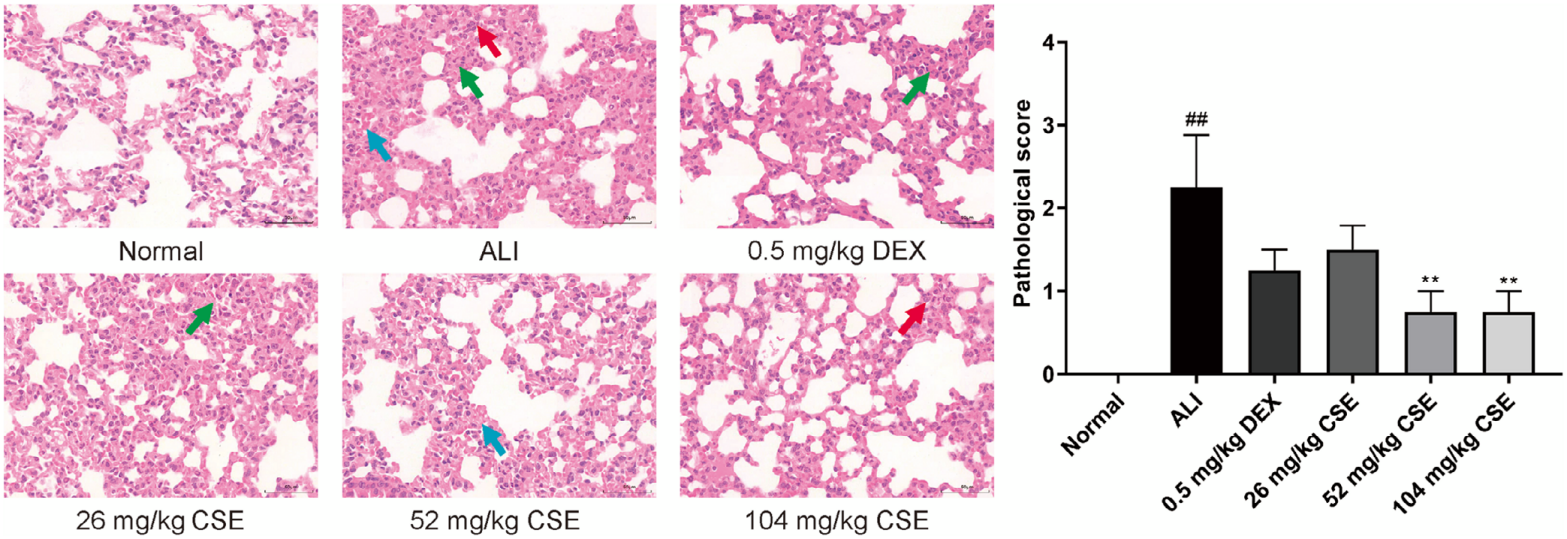
Representative HE histological sections of lung tissues (400× magnification) and scoring. Data are presented as mean ± SEM (*n* = 4).

Compared with the normal group, the lung tissues of the ALI model group exhibited vascular congestion, type II alveolar epithelial cell hyperplasia, and interstitial inflammatory cell infiltration. These lung tissue abnormalities were alleviated to varying degrees in the DEX positive control and CSE treatment groups (26, 52, and 104 mg/kg). Specifically, the 104 mg/kg CSE group exhibited reduced interstitial inflammatory cell infiltration; furthermore, no type II alveolar epithelial cell hyperplasia was observed in either the 52 or 104 mg/kg CSE groups (Figure 2). Histopathological scoring revealed that CSE at 52 and 104 mg/kg significantly decreased the pathological scores (*p* < 0.01).

### 3.3 Effects of CSE on Inflammatory Markers in the Serum and Lung Tissues

**FIGURE 3:**
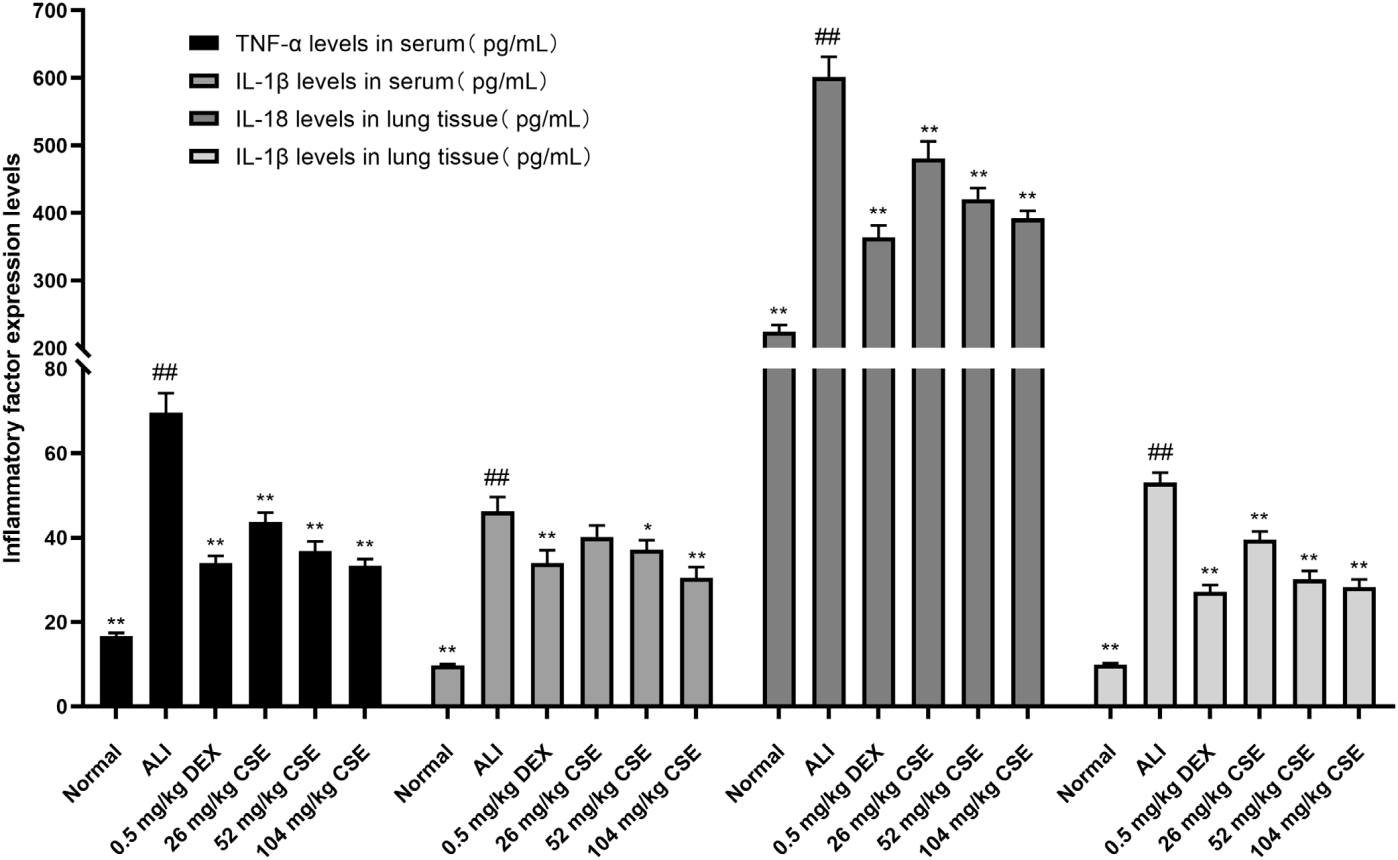
Effect of Coix seed extract (CSE) on inflammatory factors in serum and lung tissues of ALI mice. Data are presented as mean ± SEM (*n* = 10). ^#^ *p* < 0.05 versus the control group, ^##^ *p* < 0.01 versus the normal group, *p < 0.05 versus the ALI group, **p < 0.01 versus the ALI group.

Compared with the normal group, the serum levels of TNF‐α and IL‐1β, as well as the pulmonary levels of IL‐1β and IL‐18 were significantly elevated in the ALI group (*p* < 0.01). CSE administration at all doses (26, 52, and 104 mg/kg) significantly reduced the serum TNF‐α levels and the pulmonary IL‐1β and IL‐18 levels (*p* < 0.01). Additionally, CSE at 52 and 104 mg/kg significantly reduced serum IL‐1β levels (*p < 0.05*) (Figure 3).

### 3.4 LC‐MS Identification of CSE Constituents

Total ion chromatograms in both positive and negative ion modes were obtained (Figure S2). Subsequent LC‐MS analysis identified 32 CSE components that have been previously documented in the literature (Table S1).

### 3.5 “Component‐Target‐Disease” Network of CSE

**FIGURE 4:**
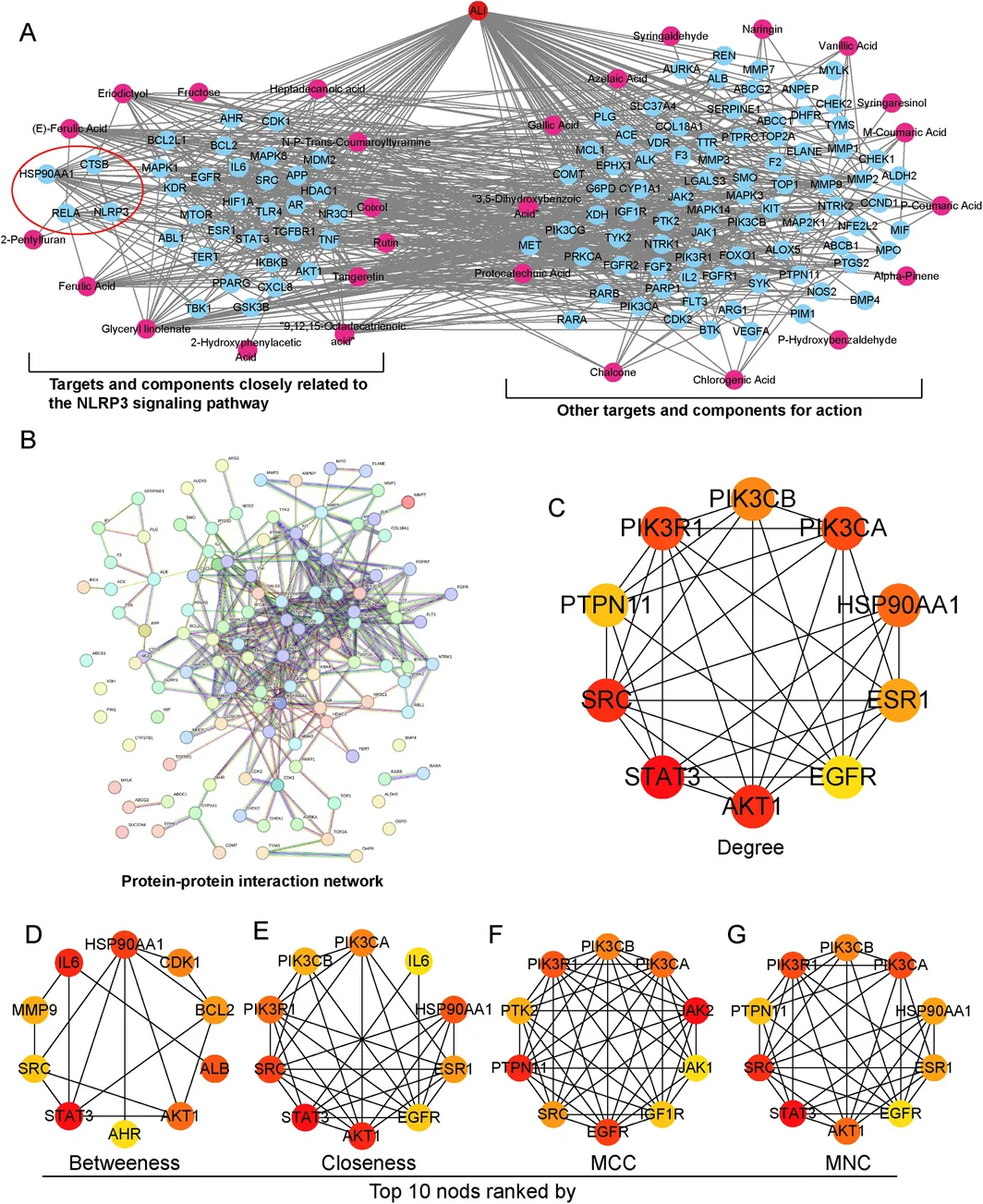
Construction and topological analysis of the “component‐target‐disease” network of Coix seed extract (CSE). (A) “Component‐target‐disease” mutual network of CSE, (B) protein–protein interaction network, and (C–G) topological analysis.

A total of 1144 potential targets were identified for CSE. By querying the GeneCards, OMIM, and TargetNet databases, 878 ALI‐associated targets were retrieved. Subsequently, the “component‐target‐disease” network for CSE was established (Figure 4A), and the corresponding PPI network was generated (Figure 4B). Based on the topological screening criteria, 37 core targets were identified, and the median values for MCC, MNC, degree, closeness and betweenness were 8.5, 3, 5, 39.175, and 42.76822, respectively (Figure 4C–G, Table S2).

### 3.6 Enrichment Analysis Results

**FIGURE 5:**
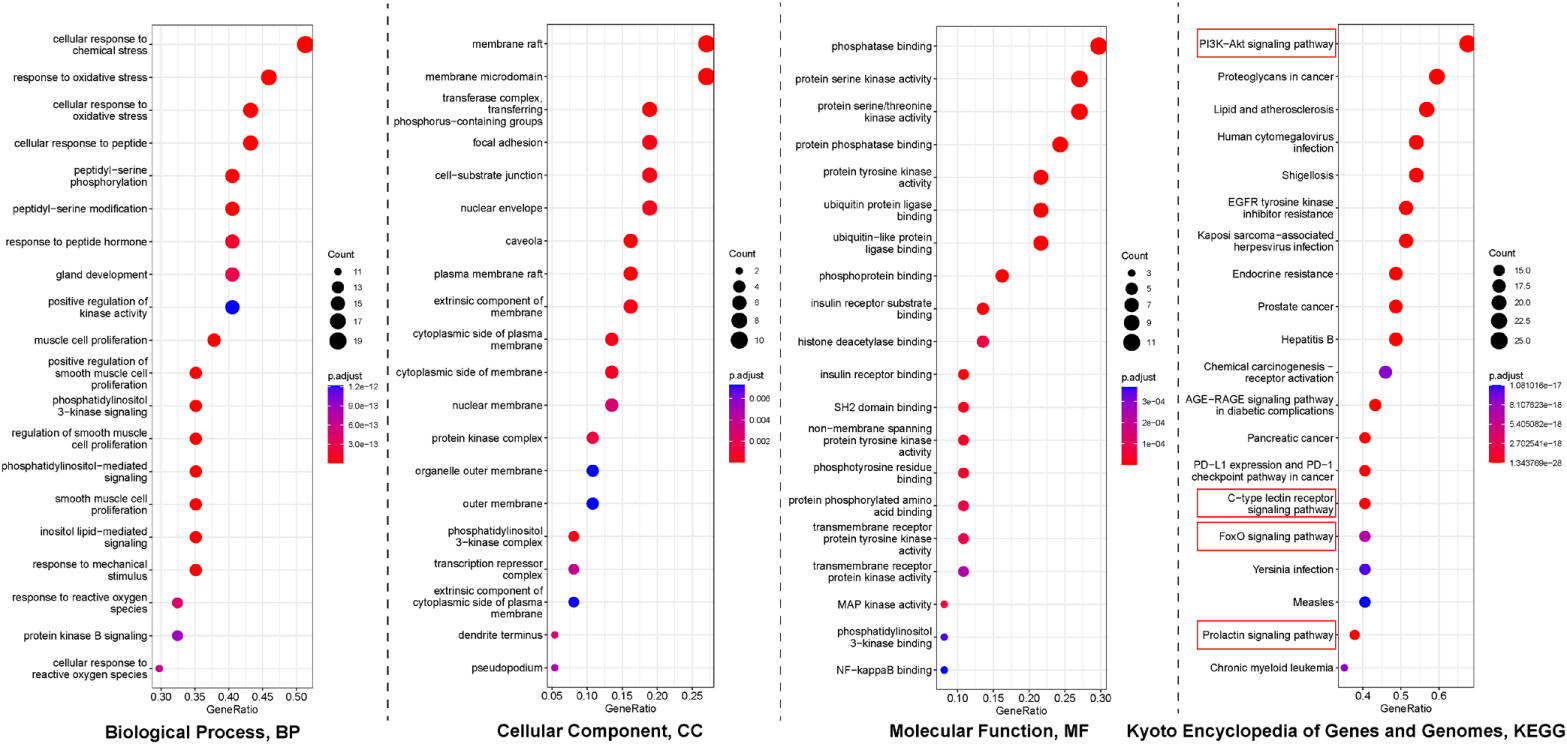
Results of GO and KEGG pathway enrichment analysis.

Biological process (BP), cellular component (CC), and molecular function (MF) terms were identified via the GO enrichment analysis (Figure 5). The primary main biological processes implicated in the protective effects of CSE against ALI included the response to oxidative stress, cellular response to oxidative stress, peptidyl‐serine phosphorylation, gland development, phosphatidylinositol‐mediated signaling, and smooth muscle cell proliferation. The signaling pathways closely related to the pathogenesis of ALI were identified via KEGG enrichment analysis, which primarily included immune‐ and inflammation‐related pathways, such as the PI3K‐Akt, prolactin, C‐type lectin receptor, FoxO, HIF‐1, Toll‐like receptor, TNF, JAK‐STAT, IL‐17, VEGF, and NOD‐like receptor signaling pathways.

### 3.7 Molecular Docking Results

STAT3 and AKT1, identified as the primary core targets based on their degree values, were selected alongside the key targets NLRP3, RELA, and CTSB for molecular docking analysis related to the NLRP3 signaling pathway. The binding affinities between the components and their respective targets ranged from −8.3 to −5.4 kcal/mol. These strong binding affinities corroborated the reliability of the “component‐target‐disease” network for CSE. Representative molecular docking models are shown in Figure S3.

### 3.8 MD Simulation Results

**FIGURE 6:**
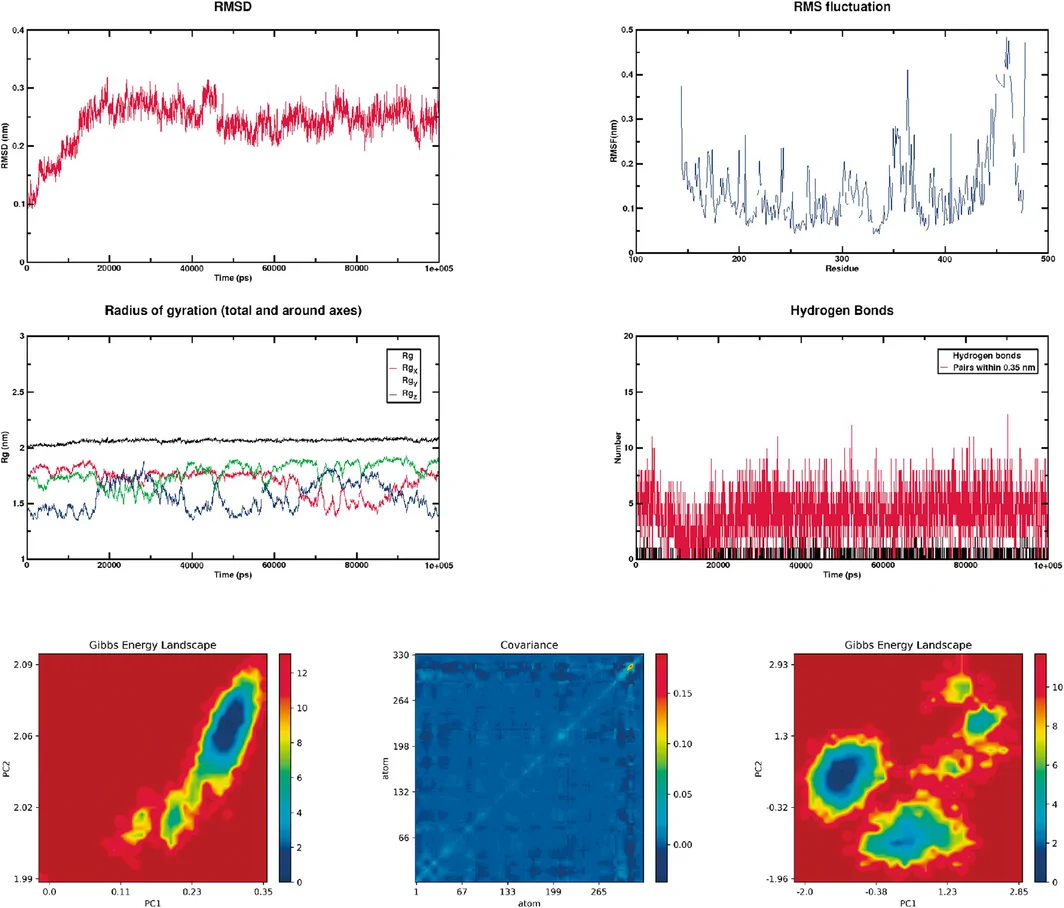
Molecular dynamics simulation results.

To evaluate the binding stability between eriodictyol and AKT1, MD simulations were performed over 100 ns. The RMSD of the protein–ligand complex backbone was calculated to assess overall conformational stability. As shown in Figure 6, the RMSD values reached equilibrium after approximately 20 ns and remained stable throughout the remainder of the simulation, with fluctuations remaining within 0.2 nm. This plateau indicates a thermodynamically stable conformation, suggesting persistent interaction between eriodictyol and AKT1. RMSF analysis revealed that most residues of AKT1 exhibited low fluctuations, indicating limited local flexibility and a rigid overall architecture (Figure 6). Furthermore, the relatively stable Rg values throughout the simulation trajectory indicated that AKT1 maintained a compact folded structure upon eriodictyol binding, without significant unfolding or expansion. Hydrogen bond analysis revealed an average of five stable hydrogen bonds maintained after 20 ns, reinforcing the specificity and affinity of the complex. Principal component analysis (PCA) and covariance analysis further supported this conformational stability, with the first two principal components accounting for the majority of the structural variance, indicating restricted conformational sampling upon ligand binding (Figure 6). Finally, Gibbs energy landscape analysis confirmed that the complex occupied a single energy minimum, corresponding to a stable binding conformation.

Collectively, the MD simulations demonstrate that eriodictyol binds stably to AKT1, maintaining the structural integrity and conformational rigidity of the protein. Given the critical role of AKT1 in regulating inflammation and cell survival, these findings provide a mechanistic basis for the therapeutic efficacy of CSE against ALI, supporting the hypothesis that CSE components exert effects via modulation of the AKT1 signaling pathway.

### 3.9 Screening of Potential Bioactive Compounds

A total of 12 compounds meeting the screening criteria were identified, including azelaic acid, (E)‐ferulic acid, eriodictyol, ferulic acid, *m*‐coumaric acid, *p*‐coumaric acid, syringaldehyde, syringaresinol, vanillic acid, chalcone, *N*‐*p* Coix seed‐trans‐coumaroyltyramine, and tangeretin.

### 3.10 Impact of CSE on the Expression of Key NLRP3 Signaling Pathway Proteins in the Lungs of ALI Mice

**FIGURE 7:**
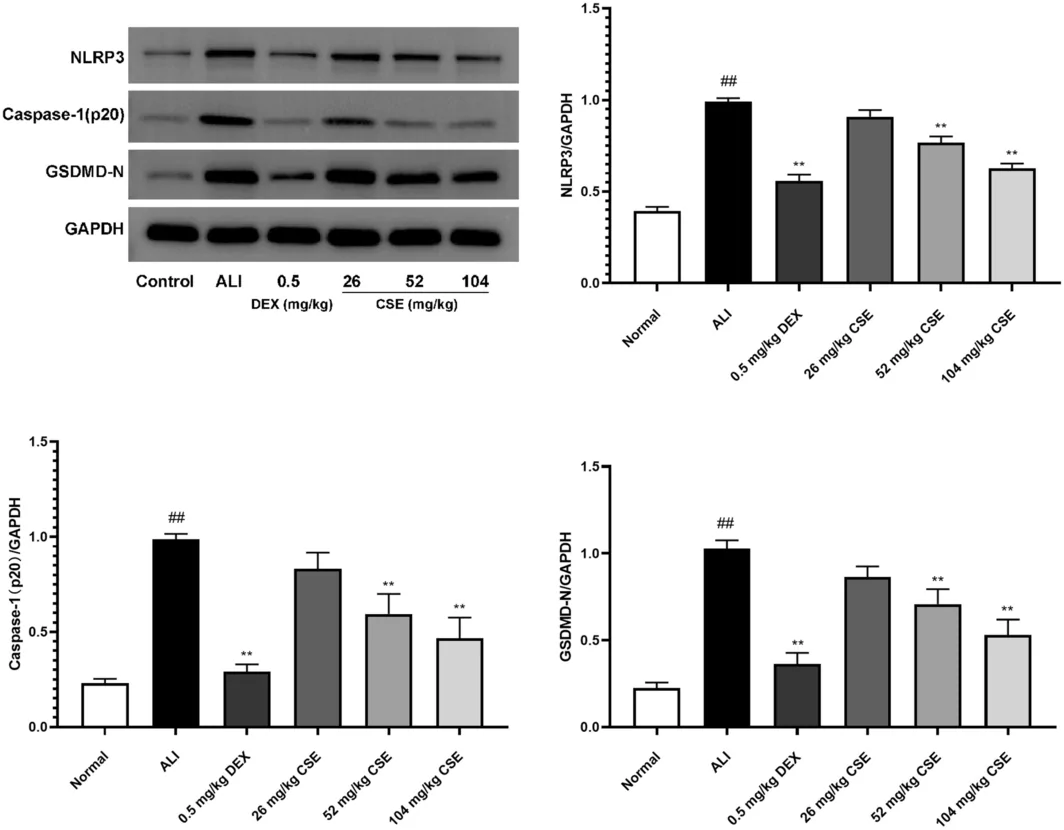
Effect of Coix seed extract (CSE) on key target proteins of NLRP3 signaling pathway in lung tissues of ALI mice. Data are presented as mean ± SEM (*n* = 4). ^#^ *p* < 0.05 versus the control group, ^##^ *p* < 0.01 versus the normal group, *p < 0.05 versus the ALI group, **p < 0.01 versus the ALI group.

Compared with the normal group, the pulmonary expression of the key proteins NLRP3, Caspase‐1, and GSDMD‐N was significantly increased in the ALI model group, indicating activation of the NLRP3 signaling pathway. Intervention with CSE at doses of 52 and 104 mg/kg significantly reduced the levels of these proteins (*p* < 0.01). Although the 26 mg/kg dose also exhibited a trend toward downregulating these proteins, the effect did not reach statistical significance (Figure 7).

## 4 Discussion

Coix seed possesses various pharmacological properties, including anti‐inflammatory, immunomodulatory, anti‐tumor, hypoglycemic, lipid‐regulating, antioxidant, and hepatoprotective effects (Chen et al. 2020; Lee et al. 2024; Sui and Xu 2024). However, prior to this study, whether CSE exerts a protective effect against ALI had remained unexplored. As a severe respiratory disease, ALI has a complicated pathogenesis and high morbidity and mortality rates, posing a significant threat to human health (Ye et al. 2025). The therapeutic efficacy of pharmacological interventions can be assessed by observing their effects on general behavioral indices, organ indices, pulmonary relative water content, and histopathological changes using the LPS‐induced murine model of ALI (Lu et al. 2024). Our findings demonstrate that CSE possesses significant therapeutic potential against ALI. Specifically, in the LPS‐induced ALI murine model, CSE improved general health status and pulmonary histopathology, while significantly reducing serum TNF‐α and IL‐1β levels, as well as pulmonary IL‐1β and IL‐18 levels. Despite significantly suppressing inflammatory cytokines and inhibiting NLRP3 activation, CSE did not significantly improve the lung wet/dry ratio. This discrepancy may reflect a temporal lag between the resolution of inflammation and the recovery of alveolar fluid clearance; the latter requires the restoration of epithelial ion channel function, which may lag behind the initial anti‐inflammatory effects. Furthermore, moderate anti‐inflammatory suppression alone may not be sufficient to reverse established barrier dysfunction. These findings suggest that anti‐inflammatory effects do not necessarily translate immediately into improvements in pulmonary edema, highlighting the need for further time‐course studies. Notably, CSE did not significantly affect the heart, liver, or kidney indices, ruling out the possibility that its pulmonary anti‐inflammatory effects were secondary to systemic organ protection. This also supports the safety of CSE at the tested doses.

LC‐MS technology is crucial for investigating the pharmacological basis of traditional Chinese medicine, enabling the efficient identification of active components (Han et al. 2025). In this study, 32 components of CSE were identified, many of which possess potent anti‐inflammatory properties, including 4‐hydroxybenzoic acid (Kou et al. 2024), azelaic acid (Mostafalou et al. 2025), chlorogenic acid (Liu et al. 2022), coixol (Zhou et al. 2024), eriodictyol (Tian et al. 2025), ferulic acid (Ermis et al. 2023), gallic acid (Kardaş et al. 2023), naringin (Gu et al. 2023), protocatechuic acid (Ding et al. 2024), rutin (Liu, Sun, et al. 2024), syringaldehyde (Tureyen et al. 2025), syringaresinol (Zhuo et al. 2022), vanillic acid (Zhou et al. 2025), and tangeretin (Peng et al. 2024). Their specific relevance to ALI represents a hypothesis generated by our network pharmacology analysis that requires future experimental validation.

Given the multicomponent, multi‐target, and multi‐pathway therapeutic nature of traditional Chinese medicine, the current research also conducted a network pharmacological analysis based on the identified components (Li et al. 2023). The “component‐target‐disease” network for CSE was successfully established, and subsequent topological analysis identified key targets, such as STAT3, AKT1, SRC, PIK3CA, PIK3R1, PIK3CB, and HSP90AA1 that are valuable for the treatment of ALI (Cao et al. 2025; Shen et al. 2024; Sun et al. 2024; Yan et al. 2024). GO and KEGG analyses revealed that CSE primarily regulates oxidative stress, kinase activity, and cell proliferation and migration in damaged tissues. Furthermore, it modulates immune‐ and inflammation‐related signaling pathways, such as the PI3K‐Akt, FoxO, C‐type lectin receptor, prolactin, Toll‐like receptor, TNF, and NOD‐like receptor signaling pathways. The Toll‐like receptor, TNF, and NOD‐like receptor signaling pathways are intimately linked to inflammation and immune regulation (Guo et al. 2024; Huang et al. 2025). C‐type lectin receptors (CLRs) function as upstream sentinels that initiate innate immune responses upon recognition of damage‐ or pathogen‐associated molecular patterns. Specifically, CLEC2 exerts protective effects (Jiang et al. 2026). Downstream of these recognition events, the PI3K‐Akt pathway serves as a pivotal signaling hub that orchestrates multiple protective responses, including the suppression of NLRP3 inflammasome‐driven pyroptosis, preservation of alveolar‐capillary barrier function, and alleviation of oxidative stress (Liu et al. 2025). Notably, the FoxO transcription factors, as downstream substrates of Akt, are intimately linked to this hub and contribute to the modulation of inflammatory and redox homeostasis (Zhang et al. 2022). It is evident that CSE exerts its therapeutic effects against ALI via multiple pathways.

Molecular docking can computationally corroborate the results of network pharmacology analyses and is widely used for the discovery of active compounds and the exploration of molecular mechanisms in traditional Chinese medicine (Yu et al. 2023). In this study, key targets (STAT3, AKT1, NLRP3, RELA, and CTSB) were selected for molecular docking analysis with their corresponding components; the results revealed strong binding affinities, preliminarily corroborating the reliability of the CSE network analysis. Furthermore, following the evaluation of GI absorption and drug‐likeness properties, 12 potential bioactive compounds were identified, six of which have been previously demonstrated to possess therapeutic efficacy against ALI: tangeretin, eriodictyol, ferulic acid, syringaresinol, *p*‐coumaric acid, and vanillic acid (Kheiry et al. 2019; Liu, Zhang, et al. 2024; Tang et al. 2022; Wang et al. 2020; Wang et al. 2025; Zhuo et al. 2022). These results indicate that the identified compounds hold significant developmental value in the treatment of ALI.

The NLRP3 inflammasome, which plays a crucial role in the pathogenesis of ALI, relies heavily on the broader NOD‐like receptor signaling pathway (Kang et al. 2022). The NLRP3 inflammasome is composed of the NLRP3 protein, the apoptosis‐associated speck‐like protein (ASC), and the precursor of the effector protein caspase‐1 (Xiao et al. 2023). The NLRP3 signaling pathway can be triggered by stimuli such as LPS, reactive oxygen species, and ATP, which in turn drive an excessive inflammatory response that precipitates lung injury (Gu et al. 2024). Furthermore, activation of the NLRP3 pathway facilitates the cleavage of GSDMD and the formation of cell membrane pores; this leads to cell swelling, rupture, and the release of inflammatory contents, ultimately disrupting the alveolar epithelial barrier and inducing pyroptotic cell death (Liu et al. 2021). Additionally, NLRP3 activation is accompanied by an increase in mitochondrial ROS, which exacerbates oxidative damage in lung tissues, induces apoptosis in endothelial cells, and promotes the formation of pulmonary edema (Liu, Zhang, et al. 2024). The NLRP3 signaling pathway also engages with various other pathways, including TLR4/NF‐κB, to exacerbate lung injury (Zhang et al. 2023). Our findings demonstrate that CSE notably reduced the expression of NLRP3, Caspase‐1, and GSDMD‐N in the lung tissues of the ALI murine model, implying that the therapeutic efficacy of CSE is mediated by the modulation of the NLRP3 signaling pathway, thereby alleviating lung inflammation and pyroptosis. The dose‐dependent effects observed for both inflammatory cytokines (Figure 3) and key protein expression levels (Figure 7) suggest that the bioactive constituents in CSE mediate their therapeutic effects against ALI via target‐specific interactions. Furthermore, this correlation indicates that the suppression of the upstream NLRP3 signaling pathway is functionally linked to the downstream attenuation of inflammatory mediator release.

Several limitations of this study should be acknowledged. First, only male mice were used. While this minimized variability associated with the estrous cycle—given that ovarian hormones modulate NLRP3 inflammasome activity—sex differences in ALI/ARDS pathogenesis and NLRP3 signaling have been documented. Thus, whether the protective effects of CSE extend to females remains unknown, and future studies incorporating both sexes or ovariectomized models are warranted. Second, the Western blot sample size (*n* = 4) is limited; although acceptable for exploratory work, larger cohorts (*n* ≥ 6) are needed for robust statistical validation. Third, the compound identifications are putative, as authentic standards were not available for all constituents; this has been noted in the Methods section. Fourth, the LPS dose (15 mg/kg, i.p.) may induce systemic effects, as discussed. Additionally, while Coix seed is traditionally regarded as non‐toxic and no acute adverse effects were observed, the long‐term safety and chronic toxicity of CSE require systematic evaluation. Finally, these findings are derived from rodent models; pharmacokinetic studies and human trials are necessary to confirm the bioavailability and clinical efficacy of CSE in at‐risk populations. Despite these limitations, the present study provides a mechanistic and pharmacological foundation for developing CSE as a functional therapeutic agent against inflammatory pulmonary conditions.

## 5 Conclusion

This study provides the first evidence of the potent therapeutic efficacy of CSE against ALI. Furthermore, 32 bioactive components of Coix seed were identified, and the core therapeutic targets such as STAT3, AKT1, SRC, and PIK3CA were delineated via network analysis. CSE appears to modulate inflammation, apoptosis, and oxidative stress by regulating multiple associated signaling pathways. Molecular docking and MD simulation analyses corroborated the reliability of these network predictions. Additionally, preliminary screening identified 12 potential bioactive compounds. In vivo validation confirmed that CSE effectively suppressed the expression of key targets (NLRP3, Caspase‐1, and GSDMD‐N) within the NLRP3 signaling pathway in the lung tissues of ALI mice. Ultimately, this study establishes a scientific basis for the therapeutic application of CSE against ALI and elucidates its underlying mechanisms of action, providing a robust pharmacological foundation for the further development and clinical utilization of Coix seed.

## Author Contributions

**Qi Yan:** writing – original draft, investigation, methodology, validation. **Yuyan Li:** investigation, writing – original draft, methodology. **Tingyan Chen:** writing – original draft, investigation, methodology. **Wenjing Liu:** investigation. **Youshuo Dong:** investigation, funding acquisition. **Zhangbo Ao:** investigation. **Hongmei Wu:** methodology. **Feng Xu:** conceptualization, investigation, funding acquisition, writing – review and editing, supervision.

## Funding

This research work was funded by the Scientific Research Project on Traditional Chinese Medicine and Ethnic Medicine of the Guizhou Provincial Administration of Traditional Chinese Medicine (Grant No.: QZYY‐2026‐077), the College Students’ Innovation and Entrepreneurship Training Program, Guizhou University of Traditional Chinese Medicine (project no. GuiZhongyi ZX He Zi [2024]44), National College Students' Innovation and Entrepreneurship Training Program (project No. 2024106620616), the Research Center for Coix Seed Resources and Development, Guizhou University of Traditional Chinese Medicine, China (project no. GuiZhongyi ZX He Zi [2024]054).

## Conflicts of Interest

The authors declare no conflicts of interest.

## Supporting information


**Figure S1:** Reproducibility study of the Coix seed extract (CSE) extraction process. The similarity between CSE batches exceeds 0.8. Reference chromatogram of Coix seed extract (A); stacked chromatograms of 10 batches of Coix seed extract (B). Chromatographic separation was performed on a Waters e2695 high‐performance liquid chromatography (HPLC) system equipped with a PDA detector. The analysis was carried out on a Diamonsil Plus C_18_ column (5 μm, 250 mm × 4.6 mm) at a column temperature of 30°C. The mobile phase consisted of acetonitrile (a) and 0.1% phosphoric acid aqueous solution (b), with a gradient elution program as follows: 0–30 min, 5%–30% a; 30–45 min, 30%–70% a; 45–70 min, 70%–100% a; 70–71 min, 100%–5% a; and 71–85 min, 5% a (isocratic). The flow rate was maintained at 1.0 mL/min, the detection wavelength was set at 254 nm, and the injection volume was 20 μL.
**Figure S2:** Total ion chromatogram of Coix seed extract (CSE). Negative (A) and positive (B) ion scanning.
**Figure S3:** Molecular docking results.
**Table S1:** Identification of components in Coix seed extract using LC‐MS.
**Table S2:** Results of core target screening.

## Data Availability

The data that support the findings of this study are available from the corresponding author upon reasonable request.
