# Supplementary material for: Coix Seed Attenuates Acute Lung Injury via NLRP3 Signaling Pathway: Efficacy, Active Components, and Mechanisms
Source: Food Sci Nutr. 2026 Sep 6;14(9):e72327. doi: 10.1002/fsn3.72327 (PMC13547748; doi:10.1002/fsn3.72327)
Supplement: Supplementary file 1 [file FSN3-14-e72327-s001.docx]

**Supporting Information**

**Coix seed attenuates acute lung injury via NLRP3 signaling pathway: Efficacy, active components, and mechanisms**

Qi Yan ^a, 1^, Yuyan Li ^a, 1^, Tingyan Chen ^a, 1^, Wenjing Liu ^a^, Youshuo Dong ^a^, Zhangbo Ao ^a^, Hongmei Wu ^a, b^, Feng Xu ^a, b*^

^a^ Guizhou University of Traditional Chinese Medicine, Department of Pharmacy, Guiyang, China

^b^ Research Center for Coix Seed Resources and Development, Guizhou University of Traditional Chinese Medicine, Guiyang, 550025, Guizhou, China

^1^ These authors contributed equally to this work.

***Correspondence**

**Feng Xu**, College of Pharmacy Guizhou University of Traditional Chinese Medicine, Huaxi District, Guiyang City, Guizhou Province, Guiyang, 550025, China.

E-mail: xf333666999@sina.com; Phone: +86 851 88308059

**Fig. S1.** Reproducibility study of the Coix seed extract (CSE) extraction process. The similarity between CSE batches exceeds 0.8. Reference chromatogram of Coix seed extract (A); stacked chromatograms of 10 batches of Coix seed extract (B). Chromatographic separation was performed on a Waters e2695 high-performance liquid chromatography (HPLC) system equipped with a PDA detector. The analysis was carried out on a Diamonsil Plus C_18_ column (5 μm, 250 mm × 4.6 mm) at a column temperature of 30 °C. The mobile phase consisted of acetonitrile (a) and 0.1% phosphoric acid aqueous solution (b), with a gradient elution program as follows: 0–30 min, 5%–30% a; 30–45 min, 30%–70% a; 45–70 min, 70%–100% a; 70–71 min, 100%–5% a; and 71–85 min, 5% a (isocratic). The flow rate was maintained at 1.0 mL/min, the detection wavelength was set at 254 nm, and the injection volume was 20 μL.

**Fig. S2.** Total ion chromatogram of Coix seed extract (CSE). negative (A) and positive (B) ion scanning.

**Fig. S3.** Molecular docking results.

**Table S1**. Identification of components in Coix seed extract using LC-MS.

| no. | name | M01 | Class I | Q1 (Da) | Ionization model | Molecular Weight (Da) | Formula |
| --- | --- | --- | --- | --- | --- | --- | --- |
| 1 | 2,4-Dihydroxybenzoic Acid | 1701044 | Polyphenols | 153 | [M-H]- | 154.1 | C7H6O4 |
| 2 | 2-Hydroxyphenylacetic Acid | 8164668 | Polyphenols | 151 | [M-H]- | 152.2 | C8H8O3 |
| 3 | 3,5-Dihydroxybenzoic Acid | 60063.46 | Organic Acid | 153 | [M-H]- | 154.1 | C7H6O4 |
| 4 | 4-Hydroxybenzoic Acid | 9860159 | Polyphenols | 137.1 | [M-H]- | 138.1 | C7H6O3 |
| 5 | Azelaic Acid | 5.1E+08 | Organic Acid | 187 | [M-H]- | 188.2 | C9H16O4 |
| 6 | Benzoic Acid | 1304218 | Organic Acid | 120.9 | [M-H]- | 122.1 | C7H6O2 |
| 7 | Chlorogenic Acid | 8029433 | Polyphenols | 353.1 | [M-H]- | 354.3 | C16H18O9 |
| 8 | Coixol | 14364629 | Others | 163.9 | [M-H]- | 165.2 | C8H7NO3 |
| 9 | (E)-Ferulic Acid | 4766982 | Phenylpropanoids | 193 | [M-H]- | 194.2 | C10H10O4 |
| 10 | Eriodictyol | 5440627 | Flavonoids | 287.1 | [M-H]- | 288.2 | C15H12O6 |
| 11 | Ferulic Acid | 10231408 | Organic Acid | 193 | [M-H]- | 194.2 | C10H10O4 |
| 12 | Gallic Acid | 256996.4 | Polyphenols | 169 | [M-H2O-H]- | 188.1 | C7H8O6 |
| 13 | M-Coumaric Acid | 38071685 | Organic Acid | 163 | [M-H]- | 164.2 | C9H8O3 |
| 14 | Naringin | 177013.7 | Flavonoids | 579.1 | [M-H]- | 580.5 | C27H32O14 |
| 15 | P-Coumaric Acid | 29171505 | Phenylpropanoids | 162.9 | [M-H]- | 164.2 | C9H8O3 |
| 16 | P-Hydroxybenzaldehyde | 1799245 | Ketones, Aldehydes,esters | 120.9 | [M-H]- | 122.1 | C7H6O2 |
| 17 | Protocatechuic Acid | 11891182 | Organic Acid | 153 | [M-H]- | 154.1 | C7H6O4 |
| 18 | Rutin | 4080.116 | Flavonoids | 609 | [M-H2O-H]- | 628.5 | C27H32O17 |
| 19 | Syringaldehyde | 394795.9 | Ketones, Aldehydes,esters | 181 | [M-H]- | 182.2 | C9H10O4 |
| 20 | Syringaresinol | 34326.53 | Lignans | 417.1 | [M-H]- | 418.4 | C22H26O8 |
| 21 | Vanillic Acid | 5127105 | Organic Acid | 167 | [M-H]- | 168.2 | C8H8O4 |
| 22 | 2-Pentylfuran | 171788.5 | Others | 139.3 | [M+H]+ | 138.2 | C9H14O |
| 23 | Alpha-Pinene | 2439.562 | Terpenoids | 137.1 | [M+H]+ | 136.2 | C10H16 |
| 24 | Chalcone | 883.6143 | Ketones, Aldehydes,esters | 209.3 | [M+H]+ | 208.3 | C15H12O |
| 25 | (-)-Limonene | 984.2196 | Terpenoids | 137.2 | [M+H]+ | 136.2 | C10H16 |
| 26 | N-P-Trans-Coumaroyltyramine | 88881.9 | Phenylpropanoids | 284 | [M+H]+ | 283.3 | C17H17NO3 |
| 27 | Tangeretin | 32969.27 | Flavonoids | 373.3 | [M+H]+ | 372.4 | C20H20O7 |
| 28 | Fructose; a-D-Furanose-form, Butyl glycoside | 9619.426 | Sugars | 281.1 | [M-H]- | 236.3 | C10H20O6 |
| 29 | Heptadecanoic acid | 65621.97 | Lipid | 315.3 | [M+FA-H]- | 270.5 | C17H34O2 |
| 30 | Glyceryl linolenate | 1012.394 | Lipid | 353.3 | [M+H]+ | 352.5 | C21H36O4 |
| 31 | Choline | 3285.442 | Others | 104.1 | [M]+ | 104.2 | C5H14NO+ |
| 32 | 9,12,15-Octadecatrienoic acid | 375.4427 | Lipid | 279.2 | [M+H]+ | 278.4 | C18H30O2 |

**Table S2**. Results of core target screening.

| No | node_name | MCC | MNC | Degree | Closeness | Betweenness |
| --- | --- | --- | --- | --- | --- | --- |
| 1 | STAT3 | 7438 | 32 | 32 | 61.03333 | 1790.23 |
| 2 | AKT1 | 894 | 23 | 25 | 56.21667 | 1078.755 |
| 3 | SRC | 57098 | 25 | 25 | 55.88333 | 669.9807 |
| 4 | PIK3R1 | 57848 | 24 | 24 | 54.21667 | 366.64 |
| 5 | PIK3CA | 57666 | 24 | 24 | 53.8 | 326.3104 |
| 6 | HSP90AA1 | 521 | 20 | 23 | 55.51667 | 1666.315 |
| 7 | PIK3CB | 57474 | 22 | 22 | 52.28333 | 242.3124 |
| 8 | ESR1 | 922 | 20 | 20 | 53.13333 | 537.7505 |
| 9 | PTPN11 | 63697 | 18 | 19 | 50.28333 | 202.1561 |
| 10 | EGFR | 58039 | 17 | 18 | 52.03333 | 347.2299 |
| 11 | IL6 | 117 | 15 | 16 | 50.6 | 1667.672 |
| 12 | JAK2 | 63720 | 16 | 16 | 49.01667 | 129.9681 |
| 13 | JAK1 | 7538 | 15 | 15 | 48.18333 | 162.518 |
| 14 | BCL2 | 200 | 12 | 14 | 50.01667 | 778.3809 |
| 15 | MAPK1 | 606 | 14 | 14 | 50.2 | 273.1633 |
| 16 | PTK2 | 56283 | 13 | 14 | 47.11667 | 79.49742 |
| 17 | TLR4 | 85 | 8 | 13 | 49.2 | 399.0495 |
| 18 | RELA | 92 | 11 | 13 | 47.2 | 268.2139 |
| 19 | TNF | 100 | 13 | 13 | 48.93333 | 238.7825 |
| 20 | MAPK3 | 222 | 13 | 13 | 48.86667 | 220.7704 |
| 21 | IGF1R | 51364 | 13 | 13 | 47.05 | 73.09533 |
| 22 | KDR | 5765 | 9 | 12 | 46.96667 | 175.6236 |
| 23 | MMP9 | 19 | 8 | 11 | 44.21667 | 777.1783 |
| 24 | MDM2 | 87 | 9 | 10 | 44.21667 | 219.8381 |
| 25 | FGF2 | 11 | 3 | 10 | 44.36667 | 168.7798 |
| 26 | MAPK8 | 38 | 10 | 10 | 46.86667 | 136.748 |
| 27 | FOXO1 | 165 | 9 | 10 | 45.13333 | 106.3472 |
| 28 | CCND1 | 42 | 9 | 9 | 45.51667 | 208.6455 |
| 29 | IKBKB | 45 | 8 | 9 | 45.38333 | 81.93351 |
| 30 | KIT | 33 | 5 | 8 | 43.11667 | 314.0445 |
| 31 | MTOR | 38 | 6 | 8 | 45.46667 | 147.5035 |
| 32 | HDAC1 | 45 | 7 | 8 | 43.13333 | 133.1214 |
| 33 | CDK1 | 9 | 4 | 7 | 40.68333 | 953.2222 |
| 34 | BCL2L1 | 16 | 5 | 7 | 43.95 | 299.6164 |
| 35 | MMP2 | 9 | 5 | 6 | 41.95 | 329.9948 |
| 36 | PTGS2 | 14 | 4 | 6 | 41.76667 | 278.5334 |
| 37 | GSK3B | 9 | 5 | 6 | 40.41667 | 44.44235 |
